# COCCOS study: Developing a transition program for adolescents with chronic conditions using Experience-Based Co-Design. A study protocol

**DOI:** 10.1371/journal.pone.0298571

**Published:** 2024-04-05

**Authors:** Natwarin Janssens, Lisa Van Wilder, Ann Van Hecke, Kim Van Hoorenbeeck, Karsten Vanden Wyngaert, Delphine De Smedt, Eva Goossens

**Affiliations:** 1 Department of Nursing and Midwifery, University of Antwerp, Antwerp, Belgium; 2 Department of Public Health and Primary Care, Ghent University, Ghent, Belgium; 3 Ghent University Hospital, Ghent, Belgium; 4 Department of Pediatrics, Antwerp University Hospital, Antwerp, Belgium; 5 Laboratory of Experimental Medicine and Pediatrics, University of Antwerp, Antwerp, Belgium; 6 Department of Public Health and Primary Care, KU Leuven, Leuven, Belgium; 7 Department of Patient Care, Antwerp University Hospital, Antwerp, Belgium; Public Library of Science, UNITED KINGDOM

## Abstract

**Background:**

During adolescence, adolescents and young adults (AYAs) are expected to transfer their care from the pediatric environment towards an adult-focused setting. To prevent an abrupt transfer of care, it is recommended to provide AYAs with chronic conditions an adequate transition program. The aim of this paper is to describe the study protocol for the development of a transition program for AYAs with common chronic conditions (COCCOS study), using the Experience-Based Co-Design (EBCD) methodology.

**Methods and analysis:**

A qualitative, participatory study is conducted in Flanders (Belgium). Study participants are AYAs (n≥15, 14–25 years old, diagnosed with type 1 diabetes, asthma, or obesity), their families, and healthcare providers (n≥15). The study is composed of eight EBCD stages: clinical site observations, in-depth interviews, trigger film, healthcare providers’ feedback event, AYAs’ feedback event, joint event, co-design workshops, and a celebration event. Photovoice will take place as a starting point of EBCD. Data will be analyzed using thematic analysis.

**Results:**

Data collection has started in January 2023 and is expected to be completed in May 2024. As of August 2023, over 15 clinical site observations have been conducted. A total of 18 AYAs, two parents, six healthcare providers have been enrolled and a total of 20 interviews have been conducted.

**Conclusion:**

Advancing transitional care is essential for tackling negative health outcomes. Applying the innovative participatory EBCD methodology will reveal key elements of transitional care for AYAs with common chronic conditions in the development of a person-centered transition program.

**Practice implications:**

Study findings will apply key elements of transitional care of AYAs with chronic conditions in the development of an adequate transition program.

## Introduction

Over the past decades, the prevalence of life-long chronic conditions in adolescents and young adults (AYA) increased substantially due to environmental and behavioral changes, improved screening and diagnosis, and medical advancements [1]. About 15 to 25% of AYAs are estimated to be affected by a chronic condition and more than 85% of them have the prospect of reaching adult age [2, 3]. These young persons are confronted with an amplified disease burden and a life-long risk for complications, requiring continuous specialized care along their life course [4, 5]. Since the needs faced by adults differ profoundly from those in younger persons, adolescents should be transferred from pediatric care towards a care setting better addressing the medical, psychosocial, and behavioral needs experienced by young adults [4, 5]. Ideally, a gradual preparation of the AYA and family, anticipating the transfer to adult care, is provided through the implementation of a transition program (TP). TPs are essential in the delivery of high-quality healthcare in AYAs with chronic conditions. These programs aim to prevent deterioration of health through the development of self-management skills, increased treatment adherence and patient empowerment, potentially leading to reduced healthcare expenses [6]. It is observed that the absence of adequate TPs results in higher symptom burden and complications, impaired patient well-being, medication non-adherence, discontinuation of care, increased emergency department and hospital use, and increased healthcare costs [7].

To date, most TPs are provided ad hoc, without clear indications on the developmental process, uptake, impact or experiences of patients and caregivers [8, 9]. Moreover, as transitional care is considered a shared responsibility between AYAs, families, pediatric and adult healthcare providers (HCP), input and support from key stakeholders in the development, implementation and evaluation of a TP is mandatory [10]. Furthermore, most TPs lack primary care involvement, despite their key role in the continuity of care for chronically ill persons [11, 12]. To date, most TPs focus on complex diseases; hence, according to the American and European Academies of Pediatrics, priority should be given to TPs in youth with less complex but more prevalent chronic conditions, such as asthma, diabetes and childhood obesity [13, 14].

To advance the quality of care for AYAs with chronic disease and to reduce variation in care across healthcare centers, the development of an evidence-based multidisciplinary transition model is considered a top priority [8, 12]. Furthermore, to achieve better TP outcomes, the involvement of people with lived experiences in the co-design of the program is recommended. “Experience-Based Co-Design” (EBCD) is a participatory approach in which AYAs, families, and HCPs act as co-designers of a care program. In essence, participants in the EBCD process act as equal partners aiming to co-design a well-accepted TP [15]. This paper describes the study protocol for developing a TP for AYAs with common chronic childhood-onset conditions (i.e., type 1 diabetes, asthma and obesity), using the EBCD methodology. The TP will be developed in close collaboration with all relevant stakeholders, both at primary and specialized care level, in Flanders (Belgium).

## Methods

### Study design

An EBCD approach is characterized by a triangulation of several qualitative methods of data collection such as observations, semi-structured interviews and focus group meetings [15]. Hence, a qualitative, explorative study design will be undertaken.

### Experience-based co-design

The EBCD-methodology is based on story-telling, starting from AYAs’ and caregivers’ experiences, identifying key touchpoints to be integrated into the TP. Using a cyclical approach, small groups of stakeholders co-design solutions and are in charge of shaping the future care program [8, 10]. The innovativeness of this methodology lies in the simultaneous involvement of AYAs, parents and HCPs throughout the entire program development. This particular approach is a critical factor in overcoming reluctance in practice [10, 15]. The EBCD toolkit and analysis techniques, as described by the Point of Care Foundation, will be consulted and applied [16]. In addition, researchers of this study were trained in the application, as described by the Point of Care Foundation.

One of the advantages of the EBCD methodology is its flexibility to adjust and adapt the method to a particular setting. Since the participation of AYAs is key in this study, additional age-appropriate features will be added to the methodology, such as the use of photovoice. Photovoice is a participatory action research method which uses photographs, taken by participants, to capture AYAs’ personal experiences, expressing their perspectives, opinions and/or feelings about the transition program [17].

### Setting and sampling

#### Setting

For partaking in the subsequent photovoice and EBCD stages, participants will be selected in collaboration with the regional University Hospitals, primary care services, community health centers, respective patient organizations for asthma and diabetes, and a specialized multidisciplinary rehabilitation center for AYAs with a diversity of chronic conditions including diabetes, asthma and obesity using a purposive sampling technique.

#### Study population

Three study populations will be selected as a sample case for the broader spectrum of common chronic childhood-onset conditions, i.e., AYAs with type 1 diabetes, asthma or obesity. Furthermore, a minimum of five, including their family members, will be enrolled for each of these conditions [18]. The proposed minimum sample size (n ≥15) will allow heterogeneity and diversity in terms of sociodemographic characteristics, ethnicity, educational level and range of experiences, but will safeguard the methodological points of attention applicable to the EBCD methodology. To support the inclusion and input of AYAs from lower socioeconomic groups, collaborations with community health centers are set up, as these centers mainly host patients with a lower socioeconomic position.

The following selection criteria will be applied for patients:

*Inclusion criteria*. AYAs aged 14–25 years (and their respective family members, with a maximum of two members per patient), diagnosed with type 1 diabetes, asthma or obesity, either facing the upcoming transfer to adult care, or already experienced with the transfer event, will be eligible.

*Exclusion criteria*. AYAs with insufficient understanding of Dutch (important for group involvement), as well as patients with neurodevelopmental, cognitive or serious mental health issues will be excluded.

Second, a sample comprising at least 15 HCPs, with experience in pediatric, adult or primary care for patients afflicted with these chronic conditions, will be recruited [18]. This multidisciplinary sample will include physicians, nurses, psychologists, dieticians, primary care providers, physiotherapists, diabetes educators, and social workers.

The following selection criteria will be applied for HCPs:

*Inclusion criteria*. HCPs with experience in the care and management of children, adolescents and/or (young) adults diagnosed with chronic conditions, working in primary or specialized care levels, will be eligible.

**Fig 1:**
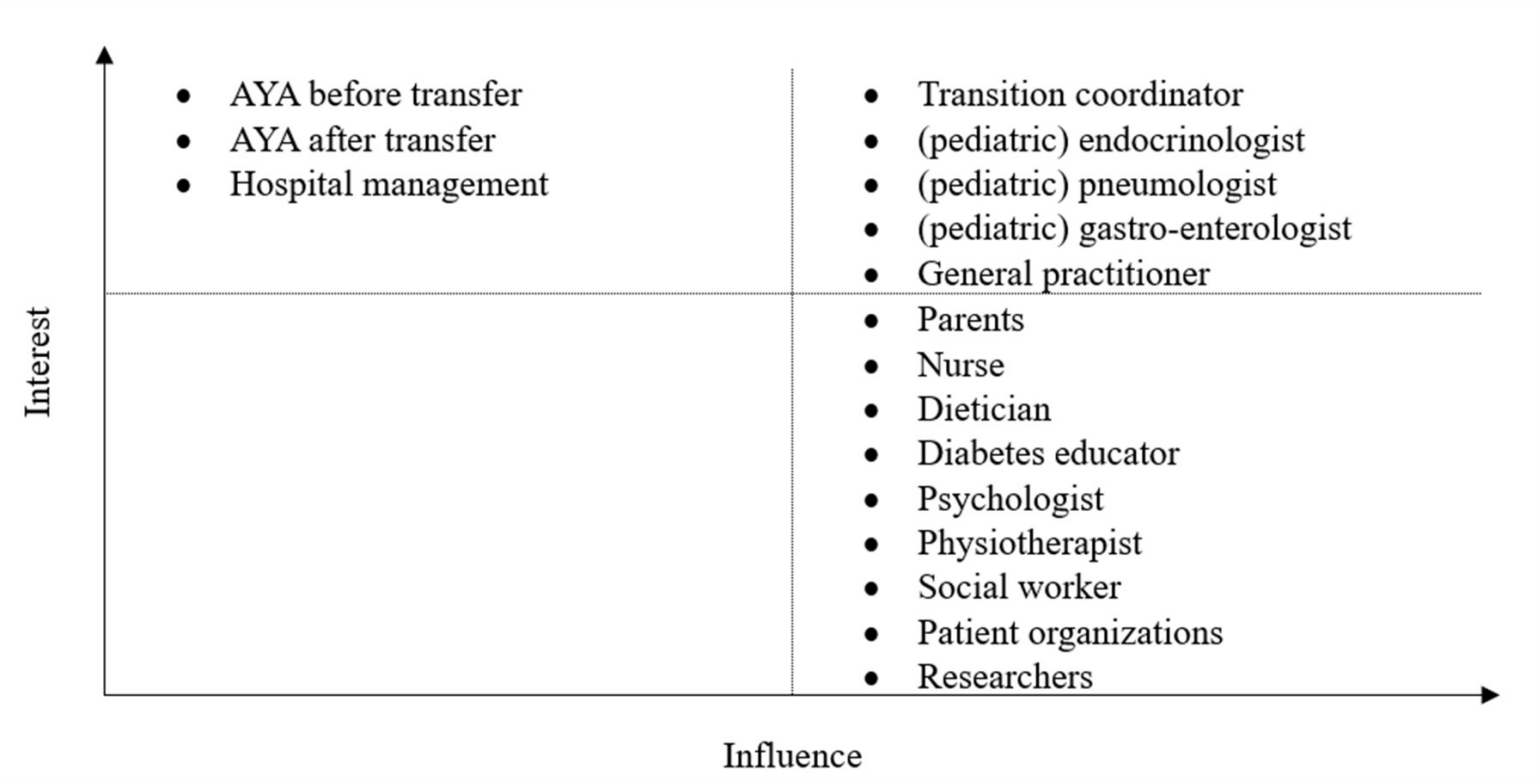
Interest-Influence classification of stakeholders.

An interest-influence classification stakeholder analysis, displayed in Fig 1, was performed to give an overview of all involved parties and their anticipated interest and influence on the study [19].

### Recruitment of participants

Primary and tertiary HCPs will be contacted through several key persons: the transition coordinator of a University Hospital, intermediary persons of the universities specialized in primary care and community health centers, and heads of department of pediatric and adult care specialized in diabetes, asthma and obesity or rehabilitation centers. An invitation to a (group) meeting will be send to those who expressed interest in the subject of the study. During these meetings, the researchers will briefly present the study and ask for HCP’s willingness to participate. Next to participation, they will be involved in the recruitment of AYA participants meeting the inclusion criteria. Clinicians will briefly explain the purpose of the study to the AYA and their family before inviting them to participate through a voluntary response sample. The AYA’s contact details will be transferred to the researcher, with a signed version of the participant informed consent. Flyers will be developed in advance to inform participants about the study, a poster will also be displayed in waiting rooms. Additional participants will be recruited when data sufficiency (i.e., alternative to data ‘saturation’ [20]) is not reached.

### Data collection

**Fig 2:**
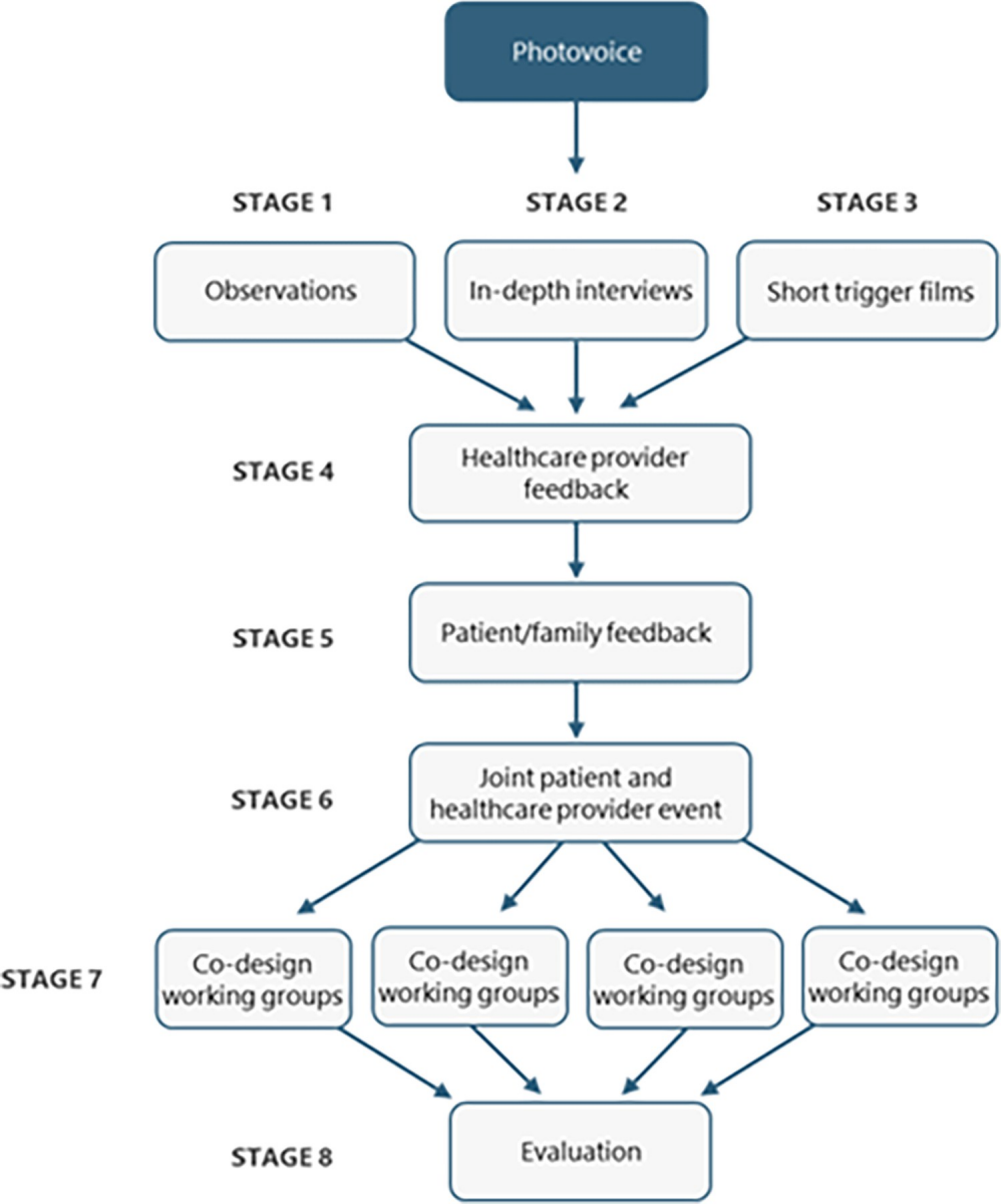
Experience-based co-design methodological steps, including photovoice.

The EBCD methodology compromises eight consecutive stages: 1) HCP’s experiences will be collected through clinical site observations; 2) in-depth interviews will be performed with AYAs, their family member(s) (videotaped) and HCPs in separate groups; 3) editing of interview videos in a short trigger film will follow subsequently; 4) HCP’s feedback event will be organized aiming to reach consensus on included items; 5) AYAs’ and family feedback event(s) will be organized showing the selected trigger film; 6) a joint AYAs’ and HCP’s event will be organized with the goal to reach consensus between all stakeholders on elements to be included in the TP; 7) running multiple co-design groups developing the TP; and 8) an evaluation event will be organized celebrating TP completion with all stakeholders (Fig 2). Not all participant groups will engage in each EBCD step. Table 1 displays the participant group(s) with the corresponding data collection steps. Photovoice will take place as a starting point for the EBCD methodology stage 2.

**Table 1:** Data collection steps with the corresponding participant groups.

	Participant group		
	Patients (and family)	Primary care providers	Tertiary care providers
**Data collection steps**			
Photovoice	X		
EBCD stage 1	X		X
EBCD stage 2	X	X	X
EBCD stage 3	X		
EBCD stage 4		X	X
EBCD stage 5	X		
EBCD stages 6, 7 and 8	X	X	X

EBCD; Experience-based co-design

### Photovoice–stage 0

AYAs will be asked to use their camera phones to document their healthcare experiences in 10–20 photographs, prior to the interview. This method will be included because it increases participant empowerment and engagement by actively involving them in a creative research project [21]. To guide the AYA, a flyer with an explanation and instructions of photovoice will be distributed among potential participants. The collected photographs will be used as a starting point for the interviews and group discussions (stage 2) planned to be performed as part of the EBCD methodology.

### EBCD stage 1

As part of EBCD stage 1, outpatient visits and primary care visits between AYAs and HCPs will be observed, to gain insight in current care practices for AYAs with diabetes, asthma or obesity including their families. Field notes (i.e., written observations) will be described in thick description before being discussed within the research team. Field notes will also be used as prompts for the interviews, next to the photovoice photographs. In total, at least five outpatient clinic and primary care visits per patient group will be observed at the University Hospitals and the rehabilitation center.

### EBCD stage 2

At least five AYAs, their family members and five HCPs per patient group will be interviewed about their experiences with the current service. The in-depth interviews will take place at the participant’s preferred location, i.e., at the hospital or the AYA’s home. For the HCPs, focus group interviews will be organized. Both interviews will adhere to a semi-structured interview guide, developed based on literature and the photovoice photographs. In consultation with the advisory board and highly experienced qualitative researchers content, validity and methodological consistency of the initial interview guide will be ensured. The interviews will last about 60 (face-to-face interviews) to 90 minutes (focus groups) and the AYA interviews will be videotaped if consent is obtained. Additionally, data are returned to participants through short conclusions of their interview to check for accuracy as a form of member checking.

### EBCD stage 3

In this stage, a 15-minute trigger film will be composed using AYA input, illustrating all key elements mentioned in EBCD stage 2 and the photovoice photographs. This film will be used to assist the feedback meetings. Participants get the possibility to see their personal recordings in advance. Footage will only be used in absence of participants opting out.

### EBCD stages 4 and 5

Both HCPs’ and AYAs’/family feedback meetings will be organized separately and moderated by the research team. These group interviews will take place at the University Hospitals and will last approximately four hours. During these meetings, the short trigger film will be shown and further discussed to identify improvement priorities for the planned intervention.

### EBCD stage 6

A heterogeneous group event will be organized with AYAs/families and HCPs. This event will last for a maximum of two hours. Five group events will be held consisting of three AYAs, their family members and three HCPs.

### EBCD stage 7

The joint priorities resulting from stage 6 will be used by the co-design working groups to develop a TP. In these working groups, AYAs/families and HCPs will work collaboratively in an iterative approach to design the TP. This TP will comprise of a generic basis allowing a modular approach with the adding-on of disease-specific modules. Both group interviews will take place at the University Hospitals.

### EBCD stage 8

The final stage includes a celebration event in which all participants will be invited to provide feedback on the co-design project. This event will be an occasion to thank all involved parties and give them closure in ending their commitment to the study. Early results and future implications will also be presented.

### Data analysis

Data collected from the in-depth (stage 2) and focus group (stage 2, 4–7) interviews will be transcribed verbatim and analyzed thematically using the software package NVivo (version 1.6.1). Data will be analyzed using content analysis, applying a multistep consecutive approach starting with an initial open coding phase, followed by axial coding and finishing with a selective coding process [22]. A constant comparison analysis will also be used within and between transcripts with the emphasis on identifying and interpreting patterns by inductively coding the data [23]. Data analysis will continue until data sufficiency has been reached. To ensure analytical rigor, at least two researchers will independently analyze the anonymized data before reflecting on the findings together. Themes and emotional touchpoints will be derived from the interviews. Field notes from the observations and focus groups will be used to support interpretation of themes emerging from the interviews.

## Ethics approval

The study entails a multicentric design, ethical approval has been provided by the Ethical Committee of Antwerp University Hospital and Ghent University Hospital (reference number: B3002022000183), Belgium.

Previous co-design studies underline the importance of guiding expectations of participants to decrease distress associated with involvement [24]. Hence, participants will receive an information letter with the aims and program for each session. To minimize the burden of study participation, participants will be reimbursed for their travel costs. All participants will receive a voucher for their time investment.

An informed consent or assent (minors) will be sought from all participants. In addition, all participants will be informed that refusal to participate or to take part but subsequently withdraw from the study, would not adversely affect their current/future care (i.e. AYAs/parents) or employment (i.e. HCPs). Data will be stored safely in a de-identified format and only accessed by project researchers on encrypted data platforms with a double authentication process, for a period of 20 years.

Study results will be disseminated through peer-reviewed publications and presented at several national and international conferences. Preliminary results will also be presented to all participants during the celebration event. A summary of the study results will be communicated to clinicians and policy makers through local, national, and international networks and events.

## Results

Data collection is anticipated to start from January 2023 and ending in May 2024. Observations have started in January 2023. The first participants were recruited in March 2023. As of August 2023, over 15 clinical site observations have been conducted at the specialized care facilities (i.e., University Hospitals and rehabilitation center) and a total of 18 AYAs, two parents and six HCPs have been enrolled in the study, 20 interviews have been conducted.

## Discussion and conclusion

### Discussion

The main strength of this study is its anticipated contribution to the healthcare organization of AYAs with type 1 diabetes, asthma and obesity. The TP will be a starting point of a tailored TP to the specific needs of these vulnerable groups. As mentioned earlier, participants are considered co-designers (i.e., ‘surrogate researchers’) of the TP and will be involved throughout each stage of the TP development. Therefore, a collective ownership between stakeholders is aimed for [25]. Furthermore, this sharing of power will be accompanied by the inclusivity of different perspectives and skills (AYAs’, families’ and HCPs’ view), equality between all participating individuals, reciprocity and strengthening of social relationships [26]. Working in close partnership with these participant groups, gives the opportunity of gaining knowledge directly from the source, hence, generating an appropriate TP [26]. Another strength is the pragmatic use of multiple participative research methods. Photovoice will be used to initiate stage 2 of the EBCD methodology, providing the opportunity to enrich the interview data [27]. In addition, methodological triangulation will be reached by using different data collection methods (i.e., photovoice, in-depth interviews, focus group interviews, observations), which increases the credibility, confirmability, and validity of research findings [28, 29]. The use of member checking (stage 2 and stage 8) will likewise increase the validity of the results. A steering committee, consisting of experts with different backgrounds, will discuss the preliminary results to ensure investigator triangulation and to monitor the achievement of milestones of the project.

Several limitations need to be considered. A potential limitation is the risk of participants’ drop-out during the EBCD process, given the substantial time investment. This potential attrition will be anticipated for, by recognizing all participants for their contribution and by showing the participants that their input is being captured in the subsequent phases of the process. AYA participants will receive a financial compensation after each meeting (e.g., a voucher) and in the end, a celebration event (stage 8) is planned to celebrate the whole process and to thank all the parties involved as recommended by the Point of Care Foundation [16]. In addition, when using an EBCD framework, equal say is key to having a widely supported outcome. However, because of the traditional roles of patients and their family versus rather ‘paternalistic’ HCPs, the former might rely on the expertise and opinion of HCPs, limiting their input in the program. Evidence of change in power relationships caused by the EBCD methodology has shown to be very scant [25]. By using small mixed groups, ensuring mutual respect, equal power relationships will be anticipated for. In addition, AYA participants will act as ‘surrogate researchers’ empowering them to not only have a voice in the process, but also an active contribution to the design of their healthcare. Another critical note is the nature of co-design research. It is often difficult to represent vulnerable groups and ‘silent voices’ among the target population since mostly ‘super’ users will participate in these studies [30]. Super users are participants who frequently participate in research studies because of their active engagement, expressiveness and clear understanding of their roles in research projects [31]. Their perspectives and experiences may have been influenced by their previous contributions to studies, therefore not entirely reflecting the target population [30].

### Conclusion

This paper describes a study protocol to co-develop a TP for AYAs with type 1 diabetes, asthma and obesity, combining photovoice and the EBCD methodology in Belgium. In a later phase, the impact of the TP prototype will be evaluated in terms of clinical effectiveness (i.e., patient-reported health outcomes), cost-effectiveness, and user experiences related to the implementation process.

## Supporting information

S1 File (PDF)

## Abbreviations

AYA: Adolescent and young adult
EBCD: Experience-Based Co-Design
HCP: Healthcare provider
TP: Transition program

## References

[1] Perrin JM, Anderson LE, Van Cleave J. The rise in chronic conditions among infants, children, and youth can be met with continued health system innovations. Health Affairs. 2014;33(12):2099–2105. doi: 10.1377/hlthaff.2014.0832 25489027

[2] Turkel S, Pao M. Late consequences of pediatric chronic illness. The Psychiatric clinics of North America. 2007;30(4):819. 17938047 10.1016/j.psc.2007.07.009 PMC2169505

[3] Jin M, An Q, Wang L. Chronic conditions in adolescents. Exp Ther Med. Jul 2017;14(1):478–482. doi: 10.3892/etm.2017.4526 28672956 PMC5488599

[4] Cooley WC, Sagerman PJ, Pediatrics AAo, Physicians AAoF. Supporting the health care transition from adolescence to adulthood in the medical home. Pediatrics. 2011;128(1):182–200. 21708806 10.1542/peds.2011-0969

[5] Pediatrics AAo Physicians AAoF, Medicine ACoP-ASoI. A consensus statement on health care transitions for young adults with special health care needs. Pediatrics. 2002;110(Supplement_3):1304–1306. 12456949

[6] Vaks Y, Bensen R, Steidtmann D, et al. Better health, less spending: redesigning the transition from pediatric to adult healthcare for youth with chronic illness. Elsevier; 2016:57–68. 10.1016/j.hjdsi.2015.09.001 PMC4805882 27001100

[7] White PH, Cooley WC, Transitions Clinical Report Authoring G, American Academy Of P, American Academy Of Family P, American College Of P. Supporting the Health Care Transition From Adolescence to Adulthood in the Medical Home. Pediatrics. Nov 2018;142(5) doi: 10.1542/peds.2018-2587 30348754

[8] Campbell F, Biggs K, Aldiss SK, et al. Transition of care for adolescents from paediatric services to adult health services. Cochrane Database of Systematic Reviews. 2016;(4). doi: 10.1002/14651858.CD009794.pub2 27128768 PMC10461324

[9] Crowley R, Wolfe I, Lock K, McKee M. Improving the transition between paediatric and adult healthcare: a systematic review. Archives of disease in childhood. 2011;96(6):548–553. doi: 10.1136/adc.2010.202473 21388969

[10] Castro EM, Malfait S, Van Regenmortel T, Van Hecke A, Sermeus W, Vanhaecht K. Co-design for implementing patient participation in hospital services: a discussion paper. Patient education and counseling. 2018;101(7):1302–1305. doi: 10.1016/j.pec.2018.03.019 29602511

[11] Scal P. Transition for youth with chronic conditions: primary care physicians’ approaches. Pediatrics. 2002;110(Supplement_3):1315–1321. 12456951

[12] Mora MA, Saarijärvi M, Moons P, Sparud-Lundin C, Bratt E-L, Goossens E. The scope of research on transfer and transition in young persons with chronic conditions. Journal of Adolescent Health. 2019;65(5):581–589. 10.1016/j.jadohealth.2019.07.014 31540780

[13] White PH, Cooley WC, Boudreau ADA, et al. Supporting the health care transition from adolescence to adulthood in the medical home. Pediatrics. 2018;142(5). doi: 10.1542/peds.2018-2587 30348754

[14] Mazur A, Dembinski L, Schrier L, Hadjipanayis A, Michaud PA. European Academy of Paediatric consensus statement on successful transition from paediatric to adult care for adolescents with chronic conditions. Acta Paediatrica. 2017;106(8):1354–1357. doi: 10.1111/apa.13901 28471516

[15] Bate P, Robert G. Experience-based design: from redesigning the system around the patient to co-designing services with the patient. BMJ quality & safety. 2006;15(5):307–310. doi: 10.1136/qshc.2005.016527 17074863 PMC2565809

[16] Point of Care Foundation. EBCD experience based co-design toolkit available at: https://www.pointofcarefoundation.org.uk/resource/experience-based-co-design-ebcd-toolkit/. 2018.

[17] Wang C, Burris MA. Photovoice: Concept, methodology, and use for participatory needs assessment. Health education & behavior. 1997;24(3):369–387. doi: 10.1177/109019819702400309 9158980

[18] Guest G, Bunce A, Johnson L. How many interviews are enough? An experiment with data saturation and variability. Field methods. 2006;18(1):59–82.

[19] Smith LW. Stakeholder analysis a pivotal practice of successful projects. 2000.

[20] LaDonna KA, Artino AR Jr, Balmer DF. Beyond the guise of saturation: rigor and qualitative interview data. The Accreditation Council for Graduate Medical Education; 2021. p. 607–611. doi: 10.4300/JGME-D-21-00752.1 34721785 PMC8527935

[21] Strack RW, Magill C, McDonagh K. Engaging youth through photovoice. Health promotion practice. 2004;5(1):49–58. doi: 10.1177/1524839903258015 14965435

[22] Hsieh H-F, Shannon SE. Three approaches to qualitative content analysis. Qualitative health research. 2005;15(9):1277–1288. doi: 10.1177/1049732305276687 16204405

[23] Glaser BG. The constant comparative method of qualitative analysis. Social problems. 1965;12(4):436–445.

[24] Tsianakas V, Robert G, Maben J, Richardson A, Dale C, Wiseman T. Implementing patient-centred cancer care: using experience-based co-design to improve patient experience in breast and lung cancer services. Supportive care in cancer. 2012;20(11):2639–2647. doi: 10.1007/s00520-012-1470-3 22544223 PMC3461206

[25] Donetto S, Pierri P, Tsianakas V, Robert G. Experience-based Co-design and Healthcare Improvement: Realizing Participatory Design in the Public Sector. The Design Journal. 2015;18(2):227–248. doi: 10.2752/175630615x14212498964312

[26] Hickey G, Denegri S, Green G, et al. Guidance on co-design research projects. 2018.

[27] Aguayo GA, Goetzinger C, Scibilia R, et al. Methods to Generate Innovative Research Ideas and Improve Patient and Public Involvement in Modern Epidemiological Research: Review, Patient Viewpoint, and Guidelines for Implementation of a Digital Cohort Study. J Med Internet Res. Dec 23 2021;23(12):e25743. doi: 10.2196/25743 34941554 PMC8738987

[28] Noble H, Heale R. Triangulation in research, with examples. Royal College of Nursing; 2019. p. 67–68. doi: 10.1136/ebnurs-2019-103145 31201209

[29] Lincoln YS, Guba EG. Establishing trustworthiness. Naturalistic inquiry. 1985;289(331):289–327.

[30] Black A, Strain K, Wallsworth C, et al. What constitutes meaningful engagement for patients and families as partners on research teams? Journal of Health Services Research & Policy. 2018;23(3):158–167. doi: 10.1177/1355819618762960 29504424 PMC6041763

[31] Moll S, Wyndham-West M, Mulvale G, et al. Are you really doing ’codesign’? Critical reflections when working with vulnerable populations. BMJ Open. Nov 3 2020;10(11):e038339. doi: 10.1136/bmjopen-2020-038339 33148733 PMC7640510

