# Supplementary material for: COCCOS study: Developing a transition program for adolescents with chronic conditions using Experience-Based Co-Design. A study protocol
Source: PLoS One. 2024 Apr 5;19(4):e0298571. doi: 10.1371/journal.pone.0298571 (PMC10997087; doi:10.1371/journal.pone.0298571)
Supplement: S1 File — (PDF) [file pone.0298571.s001.pdf]

ETHICS COMMITTEE

Prof. Dr Peter Michielsen

Secretariat

03 821 38 97

Professor Eva Goossens

Mrs Natwarin Janssens

Verpleegkunde en Vroedkunde - UAntwerpen

Project title:The COCCOS study - A CO-design for developing, implementing and evaluating a transition program for young people with chronic conditions

Project ID 3883 - Edge 002758 - BUN B3002022000183

Datum: 13/03/2023

FINAL POSITIVE ADVICE

Dear colleague

The Ethics Committee of the University Hospital Antwerp and the University Antwerp was designated the Central Ethics Committee for the delivery of the single advice for above mentioned study concerning the articles 3, 4, 6 and 7 of §4, art 11 of the law of May 7th 2004.

The answers to the letter of 21/02/2023 were discussed in the meeting of 13/03/2023.

The Ethics Committee is of the opinion that earlier remarks were sufficiently taken into account.

The following addenda were approved by the Ethics Committee, according to the ICH-GCP guidelines:

Document Type	File Name	Date	Version
GCP	Eva Goossens - GCP Certificate - UZ Leuven training - 07 May 2021	07/05/2021	1
GCP	GCP_NatwarinJanssens	04/10/2022	1
CV	CV_natwarinjanssens_ondertekend	06/10/2022	1
Insurance	Verzekering UAntwerpen 2022	07/10/2022	1
Protocol synopsis	Abstract_NL	07/10/2022	1
CV	CV_Lisa_Van_Wilder	07/10/2022	1
CV	CV Eva Goossens 21 10 2022	21/10/2022	1
Recruitment	COCCOS-poster	07/11/2022	1.0
Recruitment	Rekruteringsmail	10/11/2022	1.0
Other	Consent form for observation	12/11/2022	1
Protocol	Studieprotocol	14/11/2022	1.3
GCP	GCP_LisaVanWilder	15/11/2022	1
GCP	GCP_DelphineDeSmedt	15/11/2022	1
CV	CV DDS 2022signed	15/11/2022	1
Accompanying letter	Document 1_Brief begeleidend schrijven	16/11/2022	1
CV	CV_Luna_Antonino_22	02/12/2022	2.0
Remarks	Document 6.1_Drop-off	09/12/2022	1.2
ICF-child	Document 3.1_ICF Minderjarige participant UZ Gent (C)	22/02/2023	1.3
ICF-child	Document 3.1_ICF Minderjarige participant UZ Gent (TC)	22/02/2023	1.3

Document Type	File Name	Date	Version
ICF-child	Document 3.1_ICF Minderjarige participant UZA (C)	22/02/2023	1.3
ICF-child	Document 3.1_ICF Minderjarige participant UZA (TC)	22/02/2023	1.3
ICF	Document 3.2_ICF Experiment ouders-voogd UZA (C)	22/02/2023	1.3
ICF	Document 3.2_ICF Experiment ouders-voogd UZA (TC)	22/02/2023	1.3
ICF	Document 3.2_ICF Experiment_Ouders of voogd UZ Gent (C)	22/02/2023	1.3
ICF	Document 3.2_ICF Experiment_Ouders of voogd UZ Gent (TC)	22/02/2023	1.3
ICF	Document 3.3_ICF Meerderjarige patiënt UZA (C)	22/02/2023	1.3
ICF	Document 3.3_ICF Meerderjarige patiënt UZA (TC)	22/02/2023	1.3
ICF	Document 3.3_ICF Meerderjarige patiënten UZ Gent (C)	22/02/2023	1.3
ICF	Document 3.3_ICF Meerderjarige patiënten UZ Gent (TC)	22/02/2023	1.3
ICF	Document 3.4_ICF Zorgverleners UZ Gent (C)	22/02/2023	1.3
ICF	Document 3.4_ICF Zorgverleners UZ Gent (TC)	22/02/2023	1.3
ICF	Document 3.4_ICF ZV UZA (C)	22/02/2023	1.3
ICF	Document 3.4_ICF ZV UZA (TC)	22/02/2023	1.3
Remarks	Gegevensverwerkingsregister COCCOS project_WP1 (TC)	22/02/2023	1.2
Remarks	Gegevensverwerkingsregister COCCOS project_WP1 (C)	22/02/2023	1.2
Remarks	Document 6_Interview guide (C)	22/02/2023	1.3
Remarks	Document 6_Interview guide (TC)	22/02/2023	1.3
ICF	Document 3.5_ICF ouders-voogd UZ Gent (C)	22/02/2023	1.2
ICF	Document 3.5_ICF ouders-voogd UZA (C)	22/02/2023	1.2
Remarks	COCCOS rekruteringsflyer Photovoice	03/03/2023	1.0
Remarks	Verzekering UAntwerpen 2023	03/03/2023	1.0
Remarks	COCCOS rekruteringsflyer Photovoice	03/03/2023	1.0
Remarks	COCCOS rekruteringsbrochure	03/03/2023	1.0
Remarks	Begeleidend schrijven_COCCOS	03/03/2023	1.0

The following local Ethics Committees and the Central Ethics Committee UZA/UA give their approval of the competences of the investigators and collaborators and of the quality of the facilities:

UZ Gent

This approval is valid until one year after the above date.

Please let us know when the first participant was enrolled, when and why the study was (prematurely) stopped or never started. If the study is still running after one year, we expect a follow-up report in which any events are reported. Finally, we point out that, for studies running at the UZA, serious adverse events must be reported via the incident reporting system.

Kind regards,

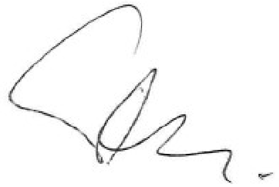

Prof. dr. Peter Michielsen

Chairman Ethics Committee UZA/UAntwerp

cc. FAGG - Research & development departement, Galileelaan 5/03, 1210 Brussel

Ethisch Comité UZ Gent - ethisch.comite@uzgent.be

Meeting Attendee Full Name	Meeting Attendee Qualifications
Prof. Dr. Bettina Blaumeiser	Physician
Emeritus Hilde Bortier	MD, PhD
Mrs Sarah Claes	Stafflmember, research dept. Antwerpen
Prof. Dr. Patrick Cras	Vice-Chair, Physician
dr Elyne De Baetselier	Nurse
Ms Ingrid De Meester	Pharmacologist
Professor Francois Eyskens	Physician
Ms Lina Fierens	Nurse
Ms Kristien Hens	Philosopher
Mr Kris Ides	Physiotherapist
Prof. Dr. Greet Ieven	Vice-Chair, Physician
Mr. Daan Kenis	Pharmacist
Dr Leon Luyten	Physician
Ms Barbara Michiels	General Practitioner
Mr Peter Michielsen	Chair, Physician
Ms Inge Michielsens	Legal
Mr Pieter Moons	Coordinator Bio- and human tissue bank
Mr Bernard Paelinck	Physician
Mr Michaël Pössel	Healthy volunteer phase 1
Ms Veerle Schoeters	Nurse
Mr Kris Smulders	Nurse
Mr Jan Van den Brande	Physician
Mr Filip Van den Eede	Physician
Mr Pieter Van Dyck	Physician
Mr Guy Van Honste	Patient Representative
Ms Dominique Van Praag	Psychologist
Mr Yves Vandermotten	Healthy voluntere phase 1
Mr Thierry Vansweevelt	Legal
Dr Michiel Voeten	Physician
Ms Griet Weyers	Pharmacist
